# Dynamic Equilibrium of CFRP-RC Square Elements under Unequal Lateral Impact

**DOI:** 10.3390/ma14133591

**Published:** 2021-06-27

**Authors:** Khalil AL-Bukhaiti, Liu Yanhui, Zhao Shichun, Hussien Abas, Dong Aoran

**Affiliations:** School of Civil Engineering, Southwest Jiaotong University, Chengdu 610031, China; khalil2020@my.swjtu.edu.cn (K.A.-B.); zscswju@home.swjtu.edu.cn (Z.S.); hus1987eng@gmail.com (H.A.); ardong.swjtu@gmail.com (D.A.)

**Keywords:** CFRPRC elements, unequal trans-lateral impact, failure mode, wrapping method, CFRP layer number

## Abstract

Building structure regularly needs reinforcement due to damage, specification requirements, and functional changes; carbon fiber reinforced polymer (CFRP) is widely used in structural reinforcement due to its high strength, lightweight, good corrosion resistance and easy construction. The regular square section reinforced concrete frame elements strengthened by CFRP material are taken as the research object. The dynamic response of CFRP to reinforced concrete elements under unequal lateral impact was discussed. This technical paper demonstrates that the test elements are subject to the bending failure mode, and the impact point and the near impact point support are severely damaged areas; the transversely wrapped elements are more abruptly broken, and the longitudinal wrapping elements and the number of wrapping layers can effectively reduce the level of damage. Analysis of the impact, deflection, and strain time history curves obtained in the test show that the wrapping mode and the number of layers have less influence on the impact force peak; the longitudinally wrapped elements and the plateau segment take longer. Dynamic equilibrium principle equation was proposed based on the experimental results. The horizontal partition plateau segment fluctuates greatly; the number of vertical wrap layers increases the plateau value. The larger the number of layers, the smaller the deflection caused by the impact. The longitudinal wrapping can effectively transmit the force.

## 1. Introduction

The application of fiber-reinforced polymer (FRP) materials in construction have increased in the past 20 years [1]. With the modernization process’s advancement and the increasing demand for railway transportation, the train’s operating density and speed have greatly increased. With the increasing number of high-speed rail stations built across China, structural safety issues have changed. This has been particularly highlighted, especially as research on the impact of train derailments on the structure’s overall safety has become increasingly important. Carbon fiber reinforced polymer (CFRP) is a type of FRPs, a polymer matrix composite material reinforced by carbon fibers. CFRP sheets’ significant advantages are pointed out as high strength, corrosion resistance, ease of implementation, and less impact on the original geometry. CFRP sheets are bonded to external faces of RC structures for multipurpose strengthening. This method can be carried out quickly, without using heavy equipment and other conventional methods (strand splices, external post-tensioning tendons, or steel plate jackets). At present, FRP composites are often used to reinforce existing concrete elements, for example, beams and columns for building structures. At present, many scholars have conducted a large number of studies on the impact resistance of structural elements. Numerous analysts researched the presentation of reinforced concrete beams and columns with FRP [2,3]. In this manner, for comparative execution levels, square or rectangular FRP confined concrete columns require more confinement than circular FRP bound concrete columns, along these lines, requiring more FRP sheet materials [4]. A cross-section could prompt a huge increment in development costs. In the previous decade, to take care of FRP confinement being less viable for columns, scholars led broad examinations on FRP confined concrete columns with square or rectangular sections. Jiang et al. [5] made a trial investigation of Fiber Reinforced Polymer (FRP)-restricted reinforced concrete (RC) columns, including distinctive bonding conditions between FRP and concrete. It was discovered that the bonding condition varieties do not significantly affect the FRP-confined RC columns’ worldwide response. Nevertheless, slipping at the bond interface harms the plastic hinge zone’s length. Related research [6] demonstrated that moisture entrance could seriously decay the long-term durability of FRP composite elements. Moreover, the temperature impact should not be neglected, particularly when the substrate is wood rather than concrete [6]. Square and rectangular columns have a wide scope of utilizations in structure development. All around, it is currently perceived that square and rectangular tubes FRP give less viable control than tube cylinders [7].

Elanchezhian et al. [8] directed three-point bending tests on three little scales prestressed concrete (PC) beams that have been harmed then strengthened with carbon fiber-reinforced polymer (CFRP) and glass fiber reinforced polymer (GFRP) directly reinforced layers. Results show that directly reinforced shear FRP can be utilized to recover and even surpass the shear limit of the intact beam. Wu et al. [9,10] verified that the corner radius ratio is directly proportional to the expansion in bound solid quality. The control viably expands the ductility of samples composed of high-strength concrete, and definitive strain increments with an increasing corner radius [11].

**Figure 1:**
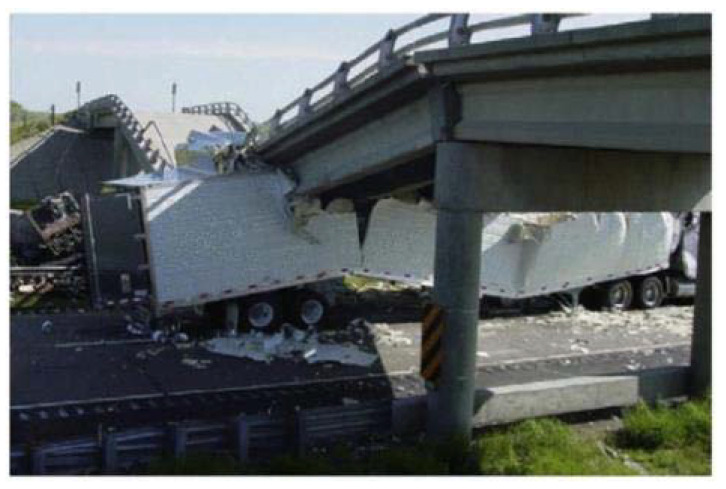
Failure of bridge due to truck collision in USA [16].

In the domestic aspect, Han Linhai [12] comprehensively expounded the stress characteristics of concrete-filled steel tube columns under different stress conditions; the Taiyuan University of Technology conducted multiple scaled concrete-filled steel column impact tests. Louw, JM [13] used 28 cantilever concrete columns and analyzed static, dynamic response characteristics when lateral load impact. SJP Richard and SHPerry [14] used a free-fall impact mounted in sleeve 70 of the concrete in the axial impact columns and described in its mechanical response. Amir Mirmiran et al. [15] used the finite element with concrete under direct impact of the finite element analysis. Through the gravity-type drop impact test on the typical structural column of the train passenger station, the dynamic response and damage characteristics of the structural column under impact can be accurately reflected, the quantitative data of the impact action can be obtained, and then the theoretical description model of the response of the element under impact can be established. This instructs the continuous resistant collapse of the overall structure. In this regard, the present study aims to improve the strengthened square columns’ impact capacity by increasing the number of layers of CFRP sheets bonded to specimens. This paper discusses the effects of the amount of CFRP sheets based on the experimental outcomes. Reinforced concrete structures often have to adjust and improve their performance during their service life. The main contributing factors are a change in use, new design standards, degradation due to corrosion in steel caused by exposure to an aggressive environment, and exposure to accidents. Additionally, impact force that is coming from the train in the metro station or shows a tractor-trailer colliding with a bridge superstructure as shown in Figure 1 [16] in Texas USA. In such circumstances, there are two possible solutions: replacement or retrofit.

The complete structure may be replaced by specific disadvantages such as the high costs of materials and labor, the most substantial environmental impact, and inconvenience due to the interruption of the structure-function, for example, traffic problems. When possible, it is best to repair the structure or update it by retrofitting. In the past decade, strong epoxy glue has led to this technique, which has great potential in developing structures. Epoxy glue involves technical connecting of steel plates or fiber-reinforced polymer plates (FRP) to the concrete surface. The plates then work together with the concrete and help carry loads. FRP can be comfortable compared to steel for several reasons. These materials have higher-end strength and lower density than steel. The installation is more convenient and temporary until the acquisition of its strength patch is not required due to low weight. The site can be formed into intricate forms and can be easily cut in situ. This work is a study of the effect of the number of CFRP layers on the square concrete elements under the impact force. This paper chose this theme because of the increasing need to strengthen the concrete structures with modern cities’ planning.

Various motivations lead to increased demand for strengthening. Given the aging infrastructure and the need to modernize to meet the stricter design requirements, structural reform and strengthening have been confirmed over the past two decades worldwide. At the same time, strengthened concrete elements have become equally important, particularly in earthquake-prone areas. Data is processed using various calculations in Microsoft Excel spreadsheets, Origin pro 9.0, CAD drawing programs, and image editing software. Information was analyzed from a qualitative and quantitative perspective. The technical paper used the American ACI 440.2R-02 [17] and the Chinese code (GB50010-2010, GB 50608, GB/T228-2010) [18] design guide for externally applied FRP systems to strengthening RC structures as reference documentation. Square reinforced concrete elements are used widely in the construction industry as internal partitioning and external boundary for RC infrastructure buildings. Considering this element in increasing the structural element’s stiffness leads to economical design, saving on the construction cost.

### 1.1. Research Objectives

This study’s main objective is to find the number of wrapped concrete layers to resist the impact forces coming from the train. The strengthening of the elements will be applied with the variable of width, spacing, and the number of the strengthening CFPR wrapping. Investigate the mechanical properties of the CFRP. In this regard, the present study investigates the parameters that can distinguish the effects of the number of layers CFRP on concrete elements and clarify the influence of CFRP layers on concrete elements to resist the impact force. In addition, it investigates the empirical design formulas following the dynamic equilibrium equations. Meanwhile, this work’s special case is present as a novelty in the impact force field, where no one in the scientific research history addressed the experimental studies under unequal lateral impact load, particularly for square concrete members with CFRP layers

### 1.2. Test Specimens Preparation

The main research was on the failure mode, impact force, and deformation of the RC element under the lateral impact of CFRP reinforcement and the dimensions. The element numbers are defined as shown in Table 1 for all of the specimens. This technical paper’s basic conditions are both ends support as fixed bearing, drop hammer mass 270 kg, Impact kinetic energy 5292 Joules, initial velocity 6.26 m/s, length of specimens = 1500 mm, element cross section = 120 × 120 mm^2^. These details were given to mimic the practical case of the derailed train that will hit the concrete element of the metro building station. The scale is 1:10, and the impact point’s position had been calculated according to the train head and the slideway height.

**Figure 2:**
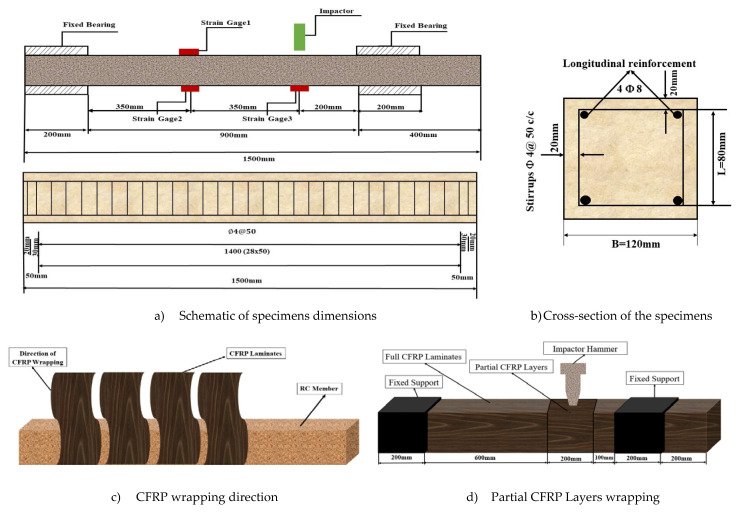
The schematic diagram for specimens.

To clarify the structural column’s dynamic response characteristics under the lateral impact, the structural column is placed in the illustrated Figure 2. The effective support length is 900 mm. The impact body under the design speed hits the structural column at 200 mm from the support edge. The three strain gauges will be shown in Figure 2a to obtain the strain time history and show element dynamic response test data.

### 1.3. Test Setup and Procedure

**Figure 3:**
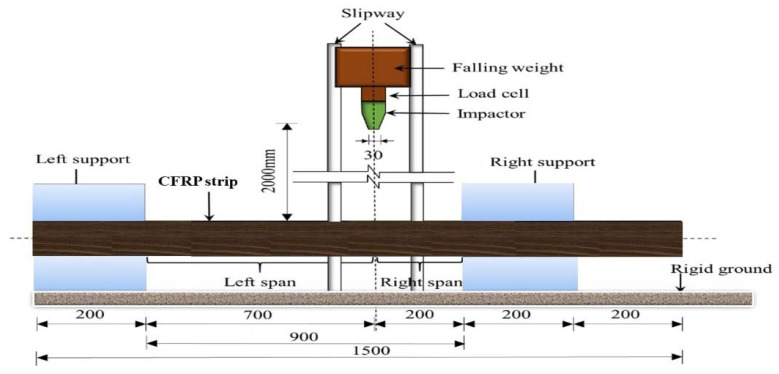
Falling-weight impact tests apparatus.

The impact test was carried out on a DHR-9401 drop hammer impact tester, the maximum height of the drop hammer guide is 13.47 m. The height of the drop hammer in this test is 2 m, and the maximum impact speed can reach 15.7 m/s. The cross-section of the drop hammer is rectangular, 80 mm long and 30 mm wide. Made of Cr with high hardness and little deformation under the impact, the weight of a single mass is 65 kg, as shown in Figure 3. In this test, the specification procedures were tested according to the ASTM’s composite standards [19]. In addition, in this test, the total impact head and mass are 270 kg. In order to accurately analyze the impact process, the impact time–history curve, impact process image, deflection time–history curve, and strain time–history curve that needs to be measured and recorded in the test are as follows:

- Time–history curve of impact force: It is measured by the mechanical sensor between the impact head and the drop hammer. The acquisition frequency is 1 MHZ.
- Impact process image: The whole process of impact deformation and failure of the element is captured by a high-speed camera placed in front of the element, with a shooting frequency of 2500 frames/second;
- Deflection time–history curve: post-processing is performed on the element’s captured image. The deformation of the element is recorded once every 0.4 ms;
- Strain time–history curve: three strain gauges were pasted on the element’s surface to measure concrete compressive strain. The first one strain gauge is 350 mm away from the bottom left support, the second one 350 mm away from the top left support, and the third on the impact point of the element’s bottom.

Drop hammer impact tests were conducted by dropping the weight from a prescribed height onto the point of 2L/9 of the element using the impact test apparatus, as shown in Figure 3. The CFRP elements are placed on the supports equipped with impact hammer load for measuring the reaction forces and are clamped at their ends using cross elements to prevent lifting off. The supports are fixed and unable to rotate freely while restraining in any direction of the elements. The weight was vertically dropped via the guide rails at 2L/9 from the elements’ right support. Here, the time history of the impact force P, strain ε, and deflection D was measured. After each test, the residual deflection was measured, and crack patterns observed on the specimens’ side surface were sketched for documentation. The impact force P and strain ε were recorded by the load cell set between the impactor and falling weight, as shown in Figure 3. The specimens’ dynamic deflection (D) was measured at 2L/9 from right support by utilizing an excel sheet for processing photos of a high-speed camera every 0.4 ms with Paint software. It was by calculating the pixel point with the white points’ coordinates that draw on the element and hammer.

## 2. Materials and Methods

### 2.1. Material Properties

#### 2.1.1. CFRP Layers

In order to measure the strength of the FRP longitudinal (Xt), transverse (Yt), and shear (S) directions, the test is following the American Society for Testing and Materials (ASTM D3039-08) [20]. Additionally, other properties for CFRP (Tokyo, Japan) are shown in Table 2. After the epoxy resin was impregnated and cured, the CFRP sheet was tested in three directions by a pull-up tester [21]. The epoxy adhesive used in the test was Araldite XH 180 (Tokyo, Japan), used for improving the properties of the concrete surface, which is in direct contact with the carbon fiber system.

#### 2.1.2. Concrete

**Figure 4:**
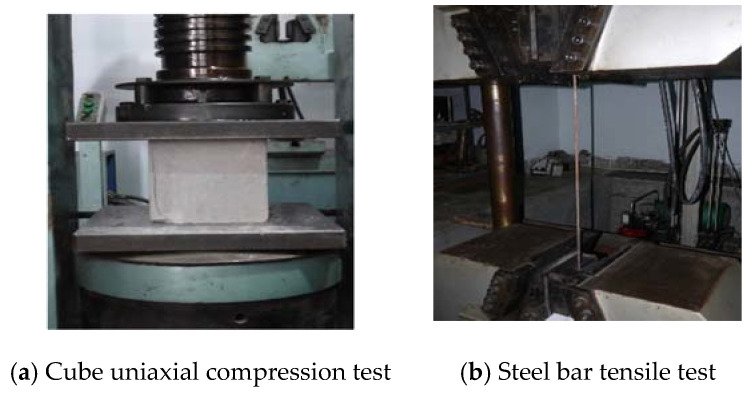
Material test of (**a**) cubic concrete, and (**b**) steel bar.

The concrete strength grade is C30. According to the Chinese Code, concrete material will be tested in the form of cubic concrete test blocks (150 × 150 × 150 mm^3^) were fabricated simultaneously as the casting elements. After 28 days of curing in water, the compressive strength test was carried out (Figure 4a), measuring the average compressive strength of concrete for 28 days $f_{c}^{\prime }$ = 43.5 MPa.

#### 2.1.3. Steel Bar

**Figure 5:**
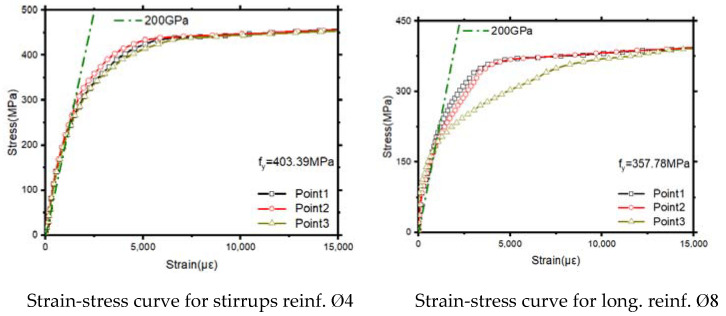
Strain–stress test curves of steel bars.

The steel bar type Φ4 (Chengdu, China) was used as transverse bars, and type Φ8 (Chengdu, China) was used as longitudinal bars (Figure 4b). Yields strength $f_{u}$ of the reinforcing bars was between 320 and 520 MPa. The strain–stress curves of steel bars are presented below in Figure 5. Maybe the paper will have an opinion in determining their yield strength that will be measured according to the “Metal Material Tensile Test Method” (GB/T228-2010) [22], as shown in Figure 5. The young’s modulus E of the reinforcement is measured through a static tensile experiment. Within the elastic limit of the reinforcement, according to Hooke’s law:

(1) $$E=\frac{\sigma }{\varepsilon }=\frac{F/A}{\Delta L/L}$$

The average ultimate tensile strength (fu) of the steel bar is measured. Parameters such as modulus (E) and elongation (δ) are detailed in Table 3.

### 2.2. Details of Test

To clarify the structural element’s dynamic response characteristics under the lateral impact, the structural element is illustrated (Figure 2a). The effective length is 900 mm. The impact body under the design speed hits the structural column 200 mm from the left support edge to obtain dynamic response test data.

#### Failure States

**Figure 6:**
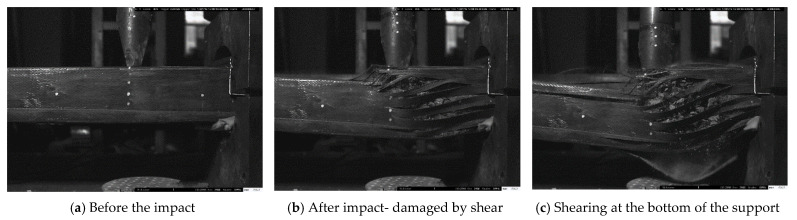
FH2-Z1 failure modes after the end of the impact.

**Figure 7:**
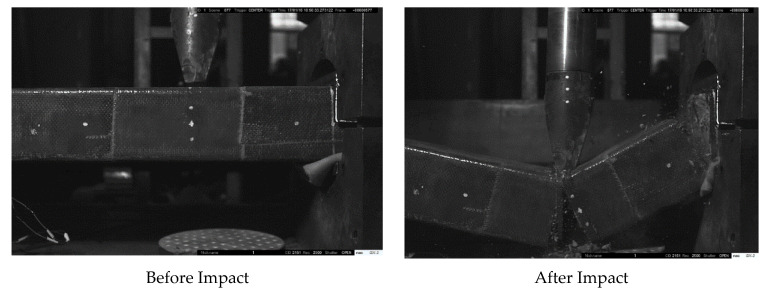
FH2-H1 failure modes after the end of the impact.

**Figure 8:**
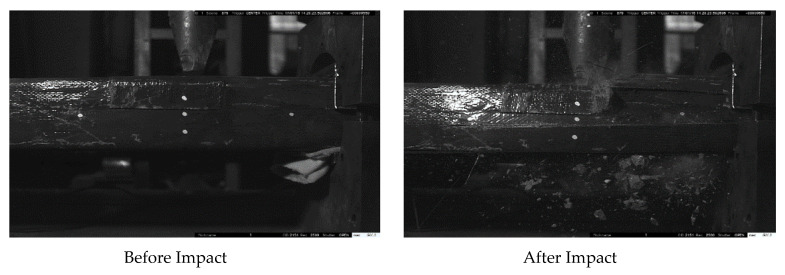
FH2-Z6 failure modes after the end of the impact.

The article shows in Figure 6, Figure 7 and Figure 8 the failure state of elements after impact as a sample for all elements. Compared with the main vertical crack on the impact section and right support section, relatively slighter cracks were found at the top of the left support section for all of the specimens. There are several vertical cracks at the upper end of the cross-section of the left support. The crack extension element’s circumambient length distribution indicates that the element is close to the left support area and is mainly subject to bending damage. The severity of the damage is not obvious compared with the right support.

It should be noted that no debonding could be observed between the CFRP and concrete, even on the crack area, as illustrated in Figure 4b for the cube uniaxial compression test. CFRP and concrete could work together under the impact load. FH2-Z1 and FH2-H1 present a completely different failure mode from Figure 6 and Figure 7. The damaged areas are concentrated on the impact point section and the right support section.

According to the static principle, the bending moment distribution of the element can be made. It can be seen that the element has a critical section at the two bearings and impact sections under the impact force; when the section is subjected to the impact, the bending moment is higher than the bending strength, that is mean the axial strain exceeds the limit state, and then the fracture begins.

As for what is in the FH2-Z6 as in Figure 8 there is no damage in, and it can be observed that the concrete inside is still right. There is no severe cracking. Only small vertical cracks appear at the upper end of the section that does not affect the whole body. FH2-Z6 has maximum resistance to impact force.

The above perceptions demonstrated that CFRP strengthening could change the RC element’s failure instrument from shear failure mode to flexural failure mode. Hence, layers could successfully keep showing up of shear failure due to more strength for RC specimens coming from CFRP layers.

## 3. Results and Discussion

### 3.1. Failure Modes

**Figure 9:**
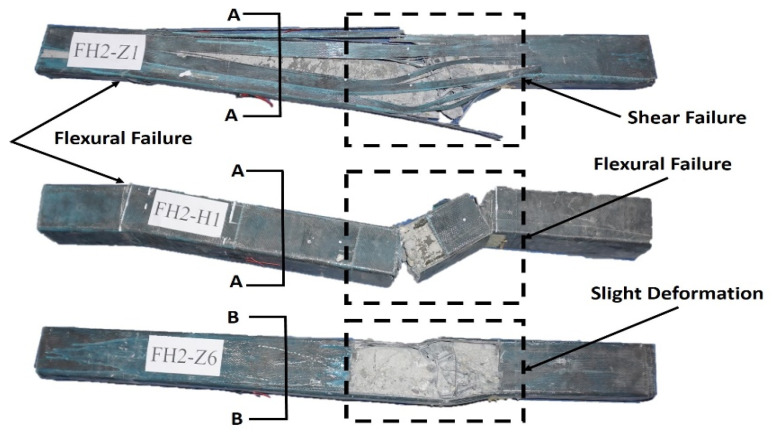
Failure modes after the end of the impact scenario for all elements.

The difference in the debonding failures of the specimens, as discussed in the failure state section, in the elements with one layer FH2-Z1 and with four layers FH2-H1confined RC elements failed by the sudden rupture of CFRP layers due to hoop tension close to the right support (Figure 6 and Figure 7). A cracking figure of epoxy rupture was first seen, followed by sudden failure modes of CFRP rupture. The failure modes of CFRP rupture have often appeared for elements with thinner CFRP layers. CFRP rupture occurred at the point of the impact hammer and near the right support in all those elements. After taking off ruptured CFRP layers and concrete cover, the elements’ examination detected that the longitudinal steel bars had buckled. The buckling deformation of steel bars near a point of impact at a distance of 700 mm from left support and at the right support was often more squeaky than that in the left support as the resistance provided by the CFRP layers is smaller at the longer sides due to the slight flexural rigidity of the CFRP layers at FH2-H1. In FH2-Z6, as Figure 8 displays, the CFRP layers’ strength resists the impact force without any failure. It can be noted that the concrete inside the area between the two vertical crack sections is still completely. There is no hard cracking, which also enables FH2-H1 to the whole body to be deformed by bending contrary to what it is in FH2-Z1. For the sake of observation, in Figure 9, the arrows are the occurrence of flexural cracks in the left support of FH2-Z1 and FH2-H1. The inside of the square dotted line is the shear failure for FH2-Z1, flexural failure for FH2-H1, and have plastic deformation. For FH2-Z6, small diagonal cracks from the impact point to the right support and can be defined as slight cracks and deformation compared to others. In the left support, there is no flexural or crack can be seen. It can also observe that the failure areas along the longitudinal direction of the specimens are limited. In other words, when the hammer hits the RC elements, all the regions near the impact zone are failed only FH2-Z6; in contrast, there is no considerable damage except the left support region for the cross-section somewhat away from the impact point (cross-section A-A). Besides, for (cross-section B-B) there is no cracking occurs. In addition, to drop height, the number of layers significantly affects the elements’ damage. This study’s experimental results revealed that the increase in the number of CFRP layers helps enhance the RC elements resistance against impact force scenario. From the comparison point of view, reducing the number of CFRP layers from four in specimen FH2-H1 to one layer in specimen FH2-Z1 leads to increased local and overall failure modes. Figure 9 indicates that the increase of the number of CFRP layers from four layers in specimen FH2-H1 to six layers in specimen FH2-Z6 greatly affects the element resistance under impact loading. The current study tendency is confirmed with the available experimental works in previous literature, which reached the same conclusion [23,24,25,26].

### 3.2. Impact Force-Time History

The impact force curve can be divided into three stages: the peak stage, the plateau phase, and the dropping section [27,28,29]. The hammer can thus be modeled as a single degree of freedom (SDOF) system. The equation of motion of the hammer according to Newton’s second law can be expressed as:

(2) $$m_{h}{\ddot{u}}_{h}+f\left(t\right)=0\;$$

where:

$m_{h}$ is hammer mass;

${\ddot{u}}_{h}$ is hammer acceleration; and

(*t*) is the recorded impact force by the load cell mounted in the hammer.

The change in hammer speed with time, when the hammer is dropped freely from a specific height, can be described as below:

(3) $${\ddot{u}}_{h}\left(t\right)=v_{0}-\int_{0}^{t}\frac{f\left(t\right)}{m_{h}}dt\;$$

where $v_{0}$ is the hammer’s initial impact velocity. Among them, the peak point occurs when the drop hammer is in contact with the element. It can be found that the force and duration of FH2-Z1, FH2-H1, and FH2-Z6 at this stage are almost the same, and the maximum peaks are 458 kN, 504 kN, and 493 kN, respectively. In the peak section, the impact force reaches the maximum value and drops immediately. This is because the force is mainly determined by the contact stiffness and the element’s inertial force. Simultaneously, the CFRP is thin and has a small density.

**Figure 10:**
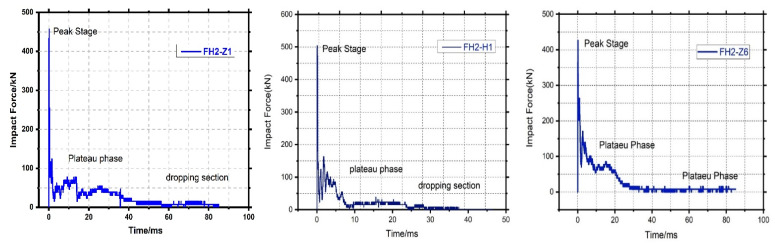
Impact time history curve.

The impact force of FH2-Z1 was significantly higher than FH2-H1 and experienced during the ascent to descent process, fluctuate is because the different deformation and failure modes of the two elements directly affect the respective stress states. In addition, this states the same as for FH2-H1. The area loses the overall force mechanism. Only the steel bars continue to be stressed, so the impact can only be maintained at a higher level of small value. After the plateau stage, the impactor and specimen have a different manner of motion [30]. The difference is that the damage of FH2-Z1 and FH2-H1 is mainly controlled by the vertical crack of the bent section. However, the contribution of the concrete to the section tension is negligible. The steel still bears most of the load, so FH2-Z1 and FH2-H1 are under the bending mechanism’s action. The load can still be continued, and the impact failure value is also stable at a higher value. In contrast, the FH2-Z6 contributes the concrete to the section tension, and the steel still bears most of the load, as shown in Figure 10.

### 3.3. Lateral Deflection Time History

**Figure 11:**
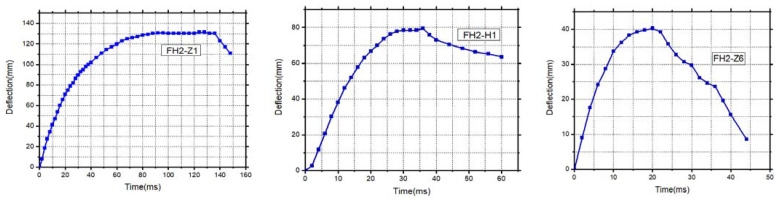
Deflection time–history curves.

Figure 11 shows the lateral deflection time history curve of each element. The lateral deflection of all types of elements shows a slight rebound after reaching the maximum value. Indicating that although all types of elements have undergone different severe damage types, they still have residual lateral directions carrying capacity. In most cases, the deflection time–history curve can be divided into two stages: (i) forced vibration stage under the impact and (ii) free vibration stage after impact. Additionally, considering that the tracing points are symmetrical about the middle span of specimens [31], CFRP-reinforced FH2-Z1, FH2-H1, and FH2-Z6 have a maximum deflection of 130, 79, and 41 mm. In contrast, FH2-Z1 has maximum lateral deflection with the longest time reaches to 125 ms, indicating that the FH2-H1 with bending failure has better resistance to deformation than the shear-destroyed FH2-Z1. However, FH2-H1 reaches the maximum lateral deflection for a more extended time, about 35 ms, because FH2-H1 weakens its impact resistance with the steel’s gradual fracture in the second stage of the impact. Even FH2-Z6 has a small lateral deflection for 20 ms, which means it has the most potent force to resist lateral deflection by six layers of CFRP. That is proof of this study’s objective to determine the number of layers of CFRP for reinforced concrete square elements.

### 3.4. Strain Time History

**Figure 12:**
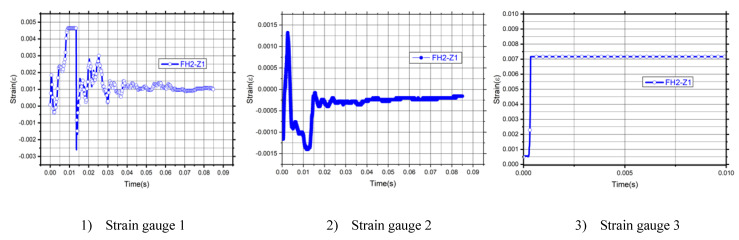

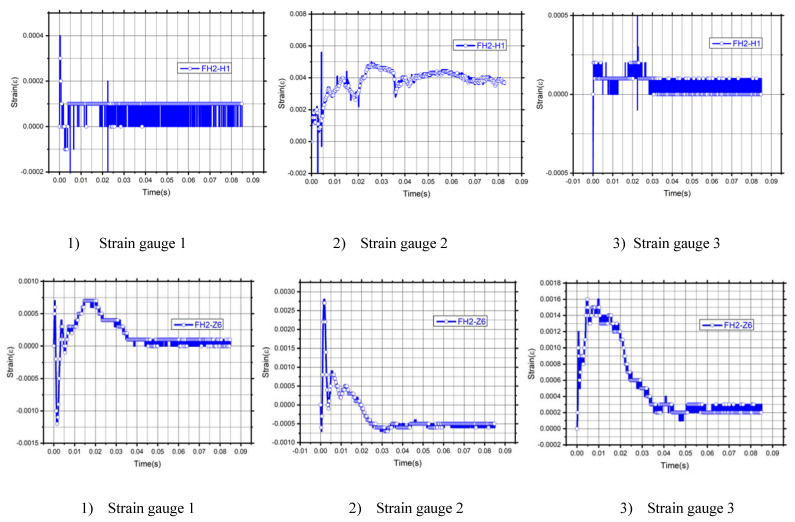
Strain time history curves analysis.

For all strain gauges, specimens showed a typical bilinear trend with a transition zone. Both the concrete and steel have a high sensitivity in strain rate effect under impact load. Three zones could be observed for the strain curves of the specimens. The strength of the materials is enhanced at a higher strain rate. Figure 12 shows the strain time–history curves analysis of each specimen. The strain time–history curves for all specimens show a slight rebound after reaching the maximum value. Although all sorts of specimens have endured different types of intense damage, they still have residual lateral directions carrying capacity. According to the first strain gauge, FH2-Z1, FH2-H1, and FH2-Z6 have a maximum strain of 0.0046, 0.0004, and 0.00074. As noted in the specimen FH2-Z1, the value fell to −0.0025 at duration 0.015 after that value returned stability in a time estimated from 0.04 to 0.085, and note that in the other specimen, FH2-H1 was the value between the decline and rise during the time 0.005 and 0.01, and then stabilized the value during the remaining time from 0.023 to 0.085 with a value between 0 to 0.001. As for the specimen FH2-Z6, the strain fell to −0.0014. During the time 0.005, it hesitated and then returned to the value 0.0054. The value hesitated and settled between the rests of the time of strain between 0 to 0.0003 at time 0.03 to 0.085. For the second strain gauge, the maximum strain values for FH2-Z1, FH2-H1, and FH2-Z6, have 0.0013, 0.0057, and 0.0027. As noted in the specimen FH2-Z1, the value fell to −0.0013 at duration 0.013 after that value returned stability in a time estimated from 0.02 to 0.085, and note that in the other specimen.

On the other hand, the third strain gauge in the point of impact on the bottom of the specimens noted for FH2-Z1, FH2-H1, and FH2-Z6 has a maximum strain of 0.007, 0.0005, and 0.0016. As noted in the specimen FH2-Z1, the curve stays at the same value along with the duration. It explains how much the specimen’s ability after failure in the impact point and there is no resistance recording after flexural strain. FH2-H1 reached the max value at 0.023, not as the other strain gauges that reached the max values at the early time, but at the impact point gauge strain value explain the ability for the enhancement multi CFRP layers to records decline and rise value between 0 and 0.00025 during the time 0.03 and 0.085. As for the specimen FH2-Z6, the strain fell to 0.00015 during the time 0.048; it hesitated and then returned to the value of 0.003 at time 0.053 to 0.085.

For the strain time history curve, one layer model’s ultimate strain had the most significant value. After that, the structure’s behavior is influenced by changing the number of CFRP layers compared with other materials. The effect of the strain time–history curve for FH2-H1 is the smallest one between all of the specimens due to the energy-absorbing by the strengthing of CFRP and the direction of wrapping for the layers. This confirmed what was published in a study by Jama 2010 et al. [32], and the same trend followed.

## 4. Dynamic Equilibrium

**Figure 13:**
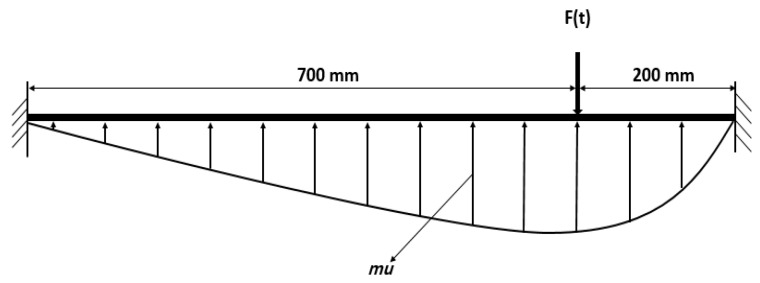
Dynamic free body diagram for the test specimen.

Suppose the drop hammer weight hits a specimen. In that case, its stiffness will resist the resultant impact force since it accelerates in the direction of impact force [33]. The acceleration produces forces of inertia equal in magnitude to the acceleration times mass. When the inertial forces’ trend is opposed to the acceleration direction, then the RC element’s points may form a state of equilibrium, which refers to (D’Alembert’s dynamic equilibrium principle) [34]. The impact event is fast such that the viscous damping force is small and can be neglected [35]. Therefore, according to this situation, a free body diagram of the dynamic equilibrium situation could be created for the test specimens, as shown in Figure 13.

According to this free body diagram, the specimen’s vertical force equilibrium can be described at any time as follows:

(4) $$P_{i}\left(t\right)+R_{l}\left(t\right)+R_{r}\left(t\right)-F\left(t\right)=0\;$$

**Figure 14:**
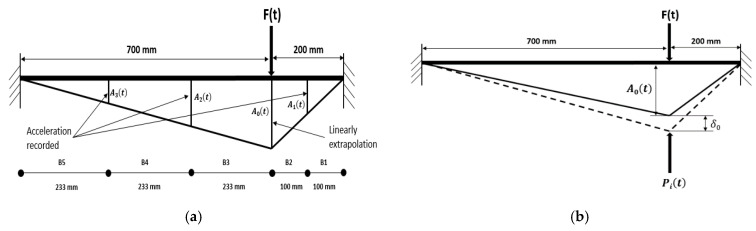
(**a**) Positions and distribution of the accelerometers, (**b**) the generalized inertial load.

If a beam segment, dx, has an acceleration A(x,t) at its center. Therefore, the inertial force acting on each element according to Newton’s laws of motion is given by [36]:

(5) dI(x,t)=mA(x,t)dx 

where $P_{i}\left(t\right),F\left(t\right),\;R_{l}\left(t\right)$ and $R_{r}\left(t\right)$ are the inertial load, impact force, and reaction forces at the left and right side, respectively. m is the mass per unit length. In this work, three accelerometers have been mounted at each specimen, as illustrated in Figure 14. If the acceleration at the contact point can achieve by linear extrapolation, and if the accelerations between the accelerometers can be achieved by linear interpolation when assuming a linear variation of the accelerations between two adjacent locations for the purpose of simplicity [37]. Therefore, the acceleration at every point along the length of the beam is known. Furthermore, when the specimen gives a virtual displacement compatible with its constraints. Furthermore, by assuming the virtual displacements at any point is proportional to the corresponding accelerations at that point, then:

(6) $$\frac{\delta_{0}}{A_{0}\left(t\right)}=\frac{\delta_{1}}{A_{1}\left(t\right)}=\frac{\delta_{2}}{A_{2}\left(t\right)}=\frac{\delta_{3}}{A_{3}\left(t\right)}\;$$

As shown in Figure 14b, *P_i_(t)* represented the generalized inertial load at the impact point 200 mm away from the right support. According to the virtual work principle, the work done by impact force *F(t)* should be equal to the virtual work done by the inertial load acting over the distributed virtual displacement.

(7) $$P_{i}\left(t\right)*\delta_{0}=\int_{0}^{l}mA\left(x,t\right)*\delta \left(x\right)dx$$

In Equation (7), *l* is the length of the specimen. On expanding Equation (7), then:

(8) $$\begin{array}{l} P_{i}\left(t\right)*\delta_{0}=[\int m*\left[A_{0}\left(t\right)-\frac{A_{0}\left(t\right)-A_{1}\left(t\right)}{B_{2}}x\right]*\left[\delta_{0}-\frac{\delta_{0}-\delta_{1}}{B_{2}}x\right]dx\\ \;+\int m*\left[A_{0}\left(t\right)-\frac{A_{0}\left(t\right)-A_{2}\left(t\right)}{B_{3}}x\right]*\left[\delta_{0}-\frac{\delta_{0}-\delta_{2}}{B_{3}}x\right]dx\\ \;+\int m*\left[A_{2}\left(t\right)-\frac{A_{2}\left(t\right)-A_{3}\left(t\right)}{B_{4}}x\right]*\left[\delta_{2}-\frac{\delta_{2}-\delta_{3}}{B_{4}}x\right]dx\\ \;+\int m*\left[A_{3}\left(t\right)-\frac{A_{3}\left(t\right)-A_{l}\left(t\right)}{B_{5}}x\right]*\left[\delta_{3}-\frac{\delta_{3}-\delta_{l}}{B_{5}}x\right]dx\\ \;+\int m*\left[A_{1}\left(t\right)-\frac{A_{2}\left(t\right)-A_{r}\left(t\right)}{B_{1}}x\right]*\left[\delta_{1}-\frac{\delta_{1}-\delta_{r}}{B_{1}}x\right]dx] \end{array}$$

*A*_1_, *A*_2_, and *A*_3_ are the values of acceleration measured by the accelerometers at three different locations on the specimen. *A*_0_ is the acceleration at the impact point obtained by linear extrapolation.$\;\delta_{0}$,$\;\delta_{1},\delta_{2},\delta_{3}$ are the virtual displacements that correspond to measured acceleration (*B_1_* + *B_3_*); furthermore, (*B_4_*) refers to the distance between the accelerometers. $A_{r}\left(t\right),\;A_{l}\left(t\right)$, and $\delta_{r},\;\delta_{l}$, are the acceleration and virtual displacement at right and left support, respectively. The accelerations and virtual displacement at the supports could be presumed to be zero. Therefore, simplification could be made to Equation (8) as follows:

(9) $$\begin{array}{l} P_{i}\left(t\right)*\delta_{0}=m[\frac{1}{3}A_{1}\left(t\right)*\delta_{0}*B_{2}+\frac{1}{3}A_{0}\left(t\right)*\delta_{0}*B_{2}+\frac{1}{3}\frac{{A^{2}}_{1}\left(t\right)}{A_{0}\left(t\right)}\delta_{0}*B_{2}+\frac{1}{3}A_{2}\left(t\right)*\delta_{0}*B_{3}\\ \;+\frac{1}{3}A_{0}\left(t\right)*\delta_{0}*B_{3}+\frac{1}{3}\frac{{A^{2}}_{2}\left(t\right)}{A_{0}\left(t\right)}\delta_{0}*B_{3}+\frac{1}{3}A_{3}\left(t\right)*\delta_{2}*B_{4}+\frac{1}{3}A_{2}\left(t\right)*\delta_{2}\\ \;*B_{4}+\frac{1}{3}\frac{{A^{2}}_{3}\left(t\right)}{A_{2}\left(t\right)}\delta_{2}*B_{4}+\frac{1}{3}A_{1}\left(t\right)*\delta_{1}*B_{1}+\frac{1}{3}A_{3}\left(t\right)*\delta_{3}*B_{5}] \end{array}$$

Utilizing Equation (6) to convey $\delta_{1},\delta_{2},and\;\delta_{3}$ in terms of $\delta_{0}$, and on cancelation $\delta_{0}$ from both sides of the equation, we get:

(10) $$\begin{array}{l} P_{i}\left(t\right)=\frac{1}{3}\;m[\frac{B_{2}}{A_{0}\left(t\right)}\left({A^{2}}_{0}\left(t\right)+{A^{2}}_{1}\left(t\right)+A_{1}\left(t\right)*A_{0}\left(t\right)\right)\\ \;+\frac{B_{3}}{A_{0}\left(t\right)}\left({A^{2}}_{2}\left(t\right)+{A^{2}}_{0}\left(t\right)+A_{2}\left(t\right)*A_{0}\left(t\right)\right)\\ \;+\frac{B_{4}}{A_{0}\left(t\right)}\left({A^{2}}_{3}\left(t\right)+{A^{2}}_{2}\left(t\right)+A_{3}\left(t\right)*A_{2}\left(t\right)\right)+\frac{B_{1}}{A_{0}\left(t\right)}{A^{2}}_{1}\left(t\right)+\frac{B_{5}}{A_{0}\left(t\right)}{A^{2}}_{3}\left(t)\right] \end{array}$$

Thus, by knowing the value of acceleration measured by the accelerometers at three different locations on the specimen and the properties of the specimens as details mentioned above. The forces of inertia can be obtained from Equation (8).

**Figure 15:**
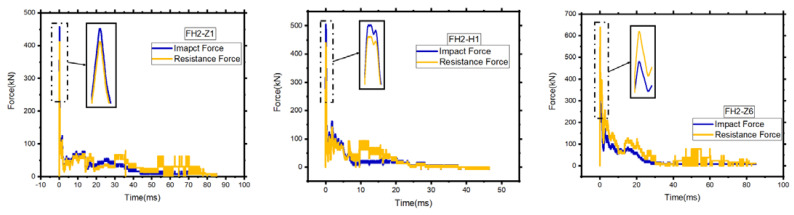
Dynamic equilibrium forces.

The dynamic equilibrium in Equation (1) for the test specimens is checked after gathering all the necessary terms. To compare, Figure 15 shows the summation of inertia and reaction forces against impact force, *F(t)*. It is noteworthy that the problems of the sampling rate and the acquisition of the high-frequency response elements influence the accuracy of dynamic forces’ results. In particular, the comparison of peak forces includes discrepancies because the initial portions of the responses had rapidly rising peaks, which were described by only a few points given the sampling rate available. Besides, the rarity of the acceleration measuring locations often brought in mistakes. Therefore, it can conclude from Figure 15. All specimens suffered from extensive damage due to the peak resisting forces were much lower than the peak impact forces.

The dynamic forces, including impact force, reaction force, and inertial force, meet the dynamic force equilibrium during the impact process. Consequently, it may make sense to use maximum reaction force rather than maximum impact force to estimate impact resistance, and that is in good agreement with what Liu et al. examined [26].

## 5. Conclusions

With a view to studying the effectiveness of the use of CFRP shear-reinforced to increase the impact resistance of CFRPRC square elements under the lateral impact, loads are studied. In this work, using three CFRPRC specimens, an unequal lateral impact test is carried out. All of the comparisons and results focus on the main idea in this study to explore the effect of the number of CFRP layers for reinforced concrete square elements. This article mainly carried out aspects of work: the impact time–history, deflection time–history, strain time–history curves, and the experimental study on the response of CFRPRC elements under lateral impact; the failure mechanism of CFRPRC elements is analyzed; and the effects of the dynamic response of the CFRPRC element were analyzed. The applicability of the proposed numerical formula was discussed compared to the experimental results for the peak impact force of the RC elements tested by Liu et al. [26]. The main conclusions obtained are as follows:

- The typical failure modes of CFRPRC elements under lateral impact are obtained. The CFRPRC element impact point and right-bearing section produce vertical cracks, section steel bar fractures, and show bending failure; the element’s failure strength is controlled by bending strength.

However, after the steel bar breaks, its impact performance decreases rapidly. The impact force decreases, and the energy consumption capacity decreases, although both elements have been severely damaged under the impact.

- CFRPRC specimens have a high impact failure value. The energy consumption is most robust before entering the falling section. However, due to the longitudinal reinforcement fracture, the failure section has a short duration. The impact performance drops rapidly after entering the descending section. The impact force is reduced, and the energy consumption capacity is weakened. The fracture of the steel bar affects the impact resistance of the CFRPRC element.
- Increasing the reinforcement ratio and adding fiber layers were shown to decrease the fiber-reinforced strain for CFRP elements and could prevent the shear failure of the RC beams.
- Based on previous dynamic equilibrium analysis, empirical design formulas following the dynamic equilibrium equations and D’Alembert’s dynamic equilibrium principle concept can be proposed: the equations involving the impact and resistance force and input impact energy.

The CFRP wrap is thus of most benefit in the scenario of lateral impact element overload. The test results presented do not enable any decisive conclusions regarding the effect of element size (cross-sectional area) on the strength increase achieved. Further tests, together with nonlinear finite element modeling to duplicate the experimental observations, would greatly enhance the current tests’ information. These preliminary tests indicate that CFRP wrapping helps increase the impact resistance of reinforced concrete square elements, particularly if a horizontal concrete layer is the first cast around the element.

- CFRP layer wrapping can be used to reinforce and strengthen the columns on the metro station and bridges, And also on the constructions for this effect on decreasing the failure and absorbing of the impact force coming from the train or vehicle trucks.

## Figures and Tables

**Table 1:** Information on CFRP specimens.

No.	Wrapping Method	Drop Height	No. of Layers	No. of Test Specimens
FH2-Z1	Vertical	2 m	1	2
FH2-H1	Horizontal (Partial 3 Layers)	2 m	1 + 3 Partial Layers	2
FH2-Z6	Horizontal	2 m	6	2

**Table 2:** CFRP performance indicators.

Material	Mechanical Parameters	
CFRP material properties	Density ρ	1800 kg/m^3^
	Calculated thickness t	1.67 mm
	Longitudinal strength Xt	3950 MPa
	Lateral strength Yt	74 MPa
	Shear strength S	108 MPa
	Longitudinal elastic modulus $E_{aa}$	2.1 × 105 MPa
	Lateral elastic modulus $E_{bb}$	1.8 × 104 MPa
	Shear modulus Gab	4657 MPa
	Longitudinal failure strain εaa	1.6%
	Lateral failure strain εbb	0.8%

**Table 3:** Rebar performance indicators.

Material	fu/MPa	E/MPa	δ	Limit Diameter	Ultimate Load
Longitudinal reinforcement	320	2.1 × 10^5^	0.21	8 mm	38 kN
Stirrup	520	2.0 × 10^5^	0.22	4 mm	9 kN

## Data Availability

The datasets used and analyzed during the current study are available from the corresponding author on reasonable request.
